# Climate warming and summer monsoon breaks drive compound dry and hot extremes in India

**DOI:** 10.1016/j.isci.2022.105377

**Published:** 2022-10-17

**Authors:** Akshay Rajeev, Shanti Shwarup Mahto, Vimal Mishra

**Affiliations:** 1 Earth Sciences, Indian Institute of Technology (IIT) Gandhinagar, Gandhinagar, Gujarat, India; 2 Civil Engineering, Indian Institute of Technology (IIT) Gandhinagar, Gandhinagar, Gujarat, India

**Keywords:** Atmospheric science, Climatology, Earth sciences, Global change

## Abstract

Considering the severe impacts of compound dry and hot extremes, we examine the primary drivers of CDHEs during the summer monsoon in India. Using ERA5 reanalysis, we show that most of the CDHEs in India occur during the droughts caused by the summer monsoon rainfall deficit. Despite a decline in the frequency of summer monsoon droughts in recent decades, increased CDHEs are mainly driven by warming and dry spells during the summer monsoon particularly in the Northeast, central northeast, and west central regions. A strong land-atmospheric coupling during droughts in the summer monsoon season leads to frequent CDHEs in the Northwest and southern peninsular regions. Furthermore, regional variations in land-atmospheric coupling cause substantial differences in the CDHE occurrence in different parts of the country. Summer monsoon rainfall variability and increased warming can pose a greater risk of compound dry and hot extremes with severe impacts on various sectors in India.

## Highlights

• CDHEs have increased in the observed period despite a decline in droughts in India
• CDHEs are largely driven by the rise in dry and warm spells during the monsoon season
• Regional variations in CDHEs are linked with the changes in land-atmospheric coupling

## Graphical abstract

**Figure:**
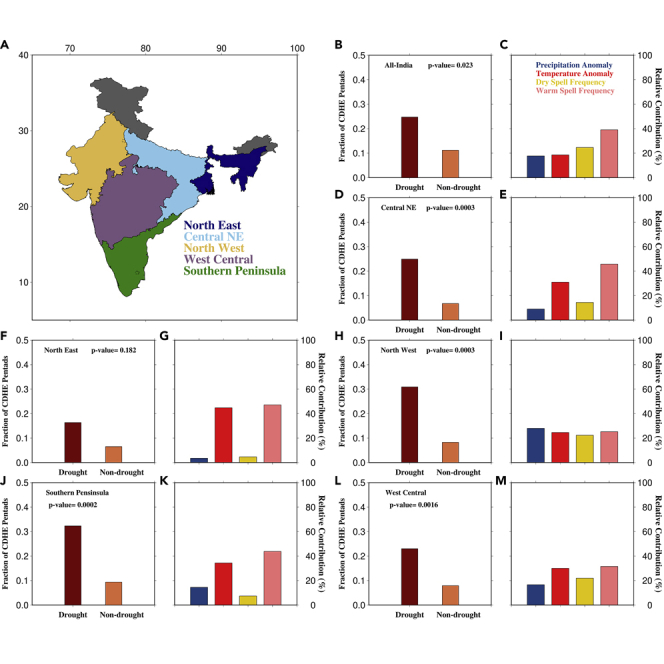

## Introduction

India has increasingly been affected by extreme weather and climate events such as droughts, floods, and heatwaves (Kumar, 2013; Panda et al., 2017; Garg and Mishra, 2019; Shah et al., 2021; Patel et al., 2022,Aadhar and Mishra, 2020, Zhang et al., 2017). Compound extremes are concurrent or consecutive occurrences of multiple extremes and their impacts are often greater than that of the individual extremes (Seneviratne et al., 2012; Leonard et al., 2014; Zscheischler et al., 2020). Due to their severity, compound dry-hot extremes (CDHEs) have been widely examined for impact assessment (Sedlmeier et al., 2018; Zscheischler et al., 2018). CDHEs can negatively impact the socioeconomic well-being of people as they can affect food security, water availability, forest mortality, and human health (Poumadère et al., 2005; Allen et al., 2010; Zscheischleret al., 2017; Mishra et al., 2020; Hettiarachchi et al., 2022,Toreti et al., 2019). In recent years, compound extremes have increased considerably in many regions worldwide (Sharma and Mujumdar, 2017; Sarhadi et al., 2018; Alizadeh et al., 2020; Mukherjee et al., 2020; Mukherjee and Mishra, 2021).

A major contributor to co-occurrence of dry and hot extremes is the land-atmospheric feedback (Seneviratne et al., 2010; Miralles et al., 2012,^2019^). For instance, the European heatwaves of 2003 were influenced by soil moisture deficit through land-atmospheric feedback (Fischer et al., 2007b). Similarly, Hauser et al. (2016) demonstrated that the dry soils over Russia led to a 6-fold rise in the heatwave risk in the region. In India, during the pre-monsoon season, hot extremes occur under extreme dry soil conditions and hence are not found to be influenced by soil moisture deficits. However, during the summer monsoon, soil moisture variability can influence temperature due to stronger land-atmospheric feedbacks (Mueller and Seneviratne, 2012; Ramarao et al., 2016). In these cases, compound extremes were caused by the land-atmospheric feedback driven by dry extremes. The land-atmospheric feedback is increasingly affected by anthropogenic warming (Seneviratne et al., 2006; Berg et al., 2016). Alizadeh et al. (2020) reported that in the USA, CDHEs were driven by meteorological droughts in the past, while anthropogenic warming has become a dominant driver during the recent period. Similarly, Vogel et al. (2021) showed that the primary cause of increased CDHE in the Mediterranean region was rising temperatures rather than decline in precipitation.

Even though CDHEs have known to significantly affect human systems (health, food, water, and energy systems), these have not been critically examined over India (Sharma and Mujumdar, 2017; Mishra et al., 2020; Dash and Maity, 2021; Guntu and Agarwal, 2021). Particularly, the role of land-atmospheric coupling on compound extremes over India remains largely unrecognized. Therefore, we aim to evaluate the changes in CDHE occurrence in India during the observed period (1950–2020). We address the three major questions: 1) How has CDHE frequency changed during the observed period and what are its major drivers? 2) How has fluctuations in monsoon rainfall and increase in climate warming affected CDHEs? 3) How crucial is the role of land-atmospheric feedback on CDHEs in India? We defined CDHEs using root zone soil moisture (∼60 cm, Mishra et al., 2018) and maximum temperature from ERA5 reanalysis to account for the land-atmospheric coupling. We estimated precipitation, maximum temperature, and soil moisture anomalies from ERA5 reanalysis for the 1950–2020 period.

## Results

### Observed changes during the summer monsoon season

**Figure 1:**
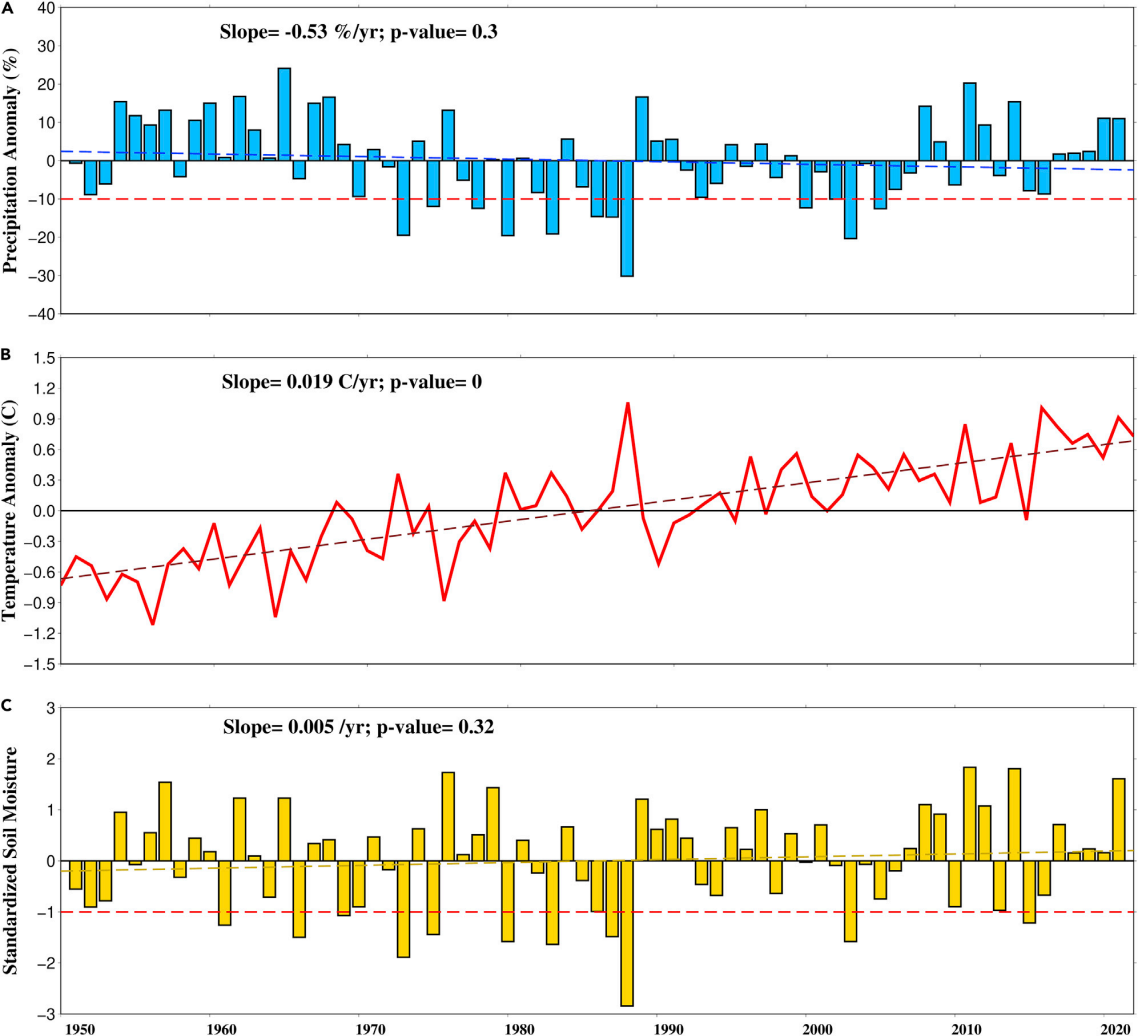
Observed changes during the summer monsoon season in India (A) Anomaly of total monsoon rainfall (%). (B) Anomaly of mean monsoon maximum temperature and (C) Standardized anomaly of mean monsoon period soil moisture. The red dashed line in A) Correspond to −10% precipitation anomaly below which we consider as drought. Similarly for C) The red dashed line corresponds to −1 standard deviation which is the threshold for drought.

First, we estimated total rainfall, maximum temperature, and soil moisture anomaly from 1950 to 2020 for the summer monsoon season (Figure 1). Since droughts occur due to summer monsoon failure, we used monsoon precipitation anomalies to identify and characterize meteorological droughts. Several years experienced precipitation anomalies less than −10% and can be classified as droughts. We identified 11 drought years while the most substantial rainfall deficit occurred during 1987 with rainfall deficit of −30%. We find a gradual decline in the summer monsoon rainfall over India during 1950–2020 (Figure S1), which can be attributed to anthropogenic factors such as warming in the Indian ocean, agricultural intensification, and anthropogenic aerosols (Bollasina et al., 2011; Mishra et al., 2012; Roxy et al., 2015). The decline in the summer monsoon precipitation is also linked with the increase in dry spells (Rajeevan et al., 2010) and may affect the frequency of compound dry-hot extremes.

Air temperature during the summer monsoon season increased in the observed period of 1950–2020 (Figures 1B and S1). The mean monsoon temperature anomaly remains negative in most years before 1980. However, after 1980, temperature during the summer monsoon showed positive anomalies and increased continuously (Figure 1B). Driven primarily by anthropogenic warming, this rise in temperature could also be modulated by summer monsoon rainfall variability as we find a decline in monsoon precipitation over India (Figures 1A and S1). Recent studies reported that CDHEs are increasingly driven by temperature rather than dry extremes (Sarhadi et al., 2018; Alizadeh et al., 2020). Moreover, soil moisture plays a role in intensifying and propagating temperature extremes during CDHE events because of robust land-atmospheric feedbacks during CDHEs (Miralles et al., 2019).

Considering the importance of land-atmospheric coupling in CDHEs, we use soil moisture to characterize droughts. We estimated SSI for the monsoon season and defined drought years as SSI less than −1 (Figure 1C). Like meteorological droughts, we identified 11 agricultural droughts (based on SSI) in the observed period, with the most severe drought occurring in 1987 (SSI = −2.8). Standardized indices based on soil moisture and precipitation captured droughts during the observed period like the other methods/indices used in the previous studies (Kumar, 2013, Shah and Mishra, 2020). For instance, 1966, 1987, and 2002 droughts were also identified using Standardized Precipitation Evapotranspiration Index (SPEI) by Kumar et al. (2013) and using Integrated Drought Index (IDI) by Shah and Mishra, (2020). The summer monsoon SSI has increased despite a decline in the monsoon precipitation. Anthropogenic factors may be a driver of this contrast in the summer monsoon precipitation and soil moisture over India. For instance, Niyogi et al. (2010) show that agricultural intensification and irrigation during the pre-monsoon season may contribute to this rise of monsoon season SSI. Similarly, Ambika and Mishra, (2019) showed the influence of intensive irrigation over the Indo-Gangetic plain on cooling primarily due to enhanced evapotranspiration. Thus, precipitation and soil moisture trends may not be consistent in the regions that are intensively irrigated.

**Figure 2:**
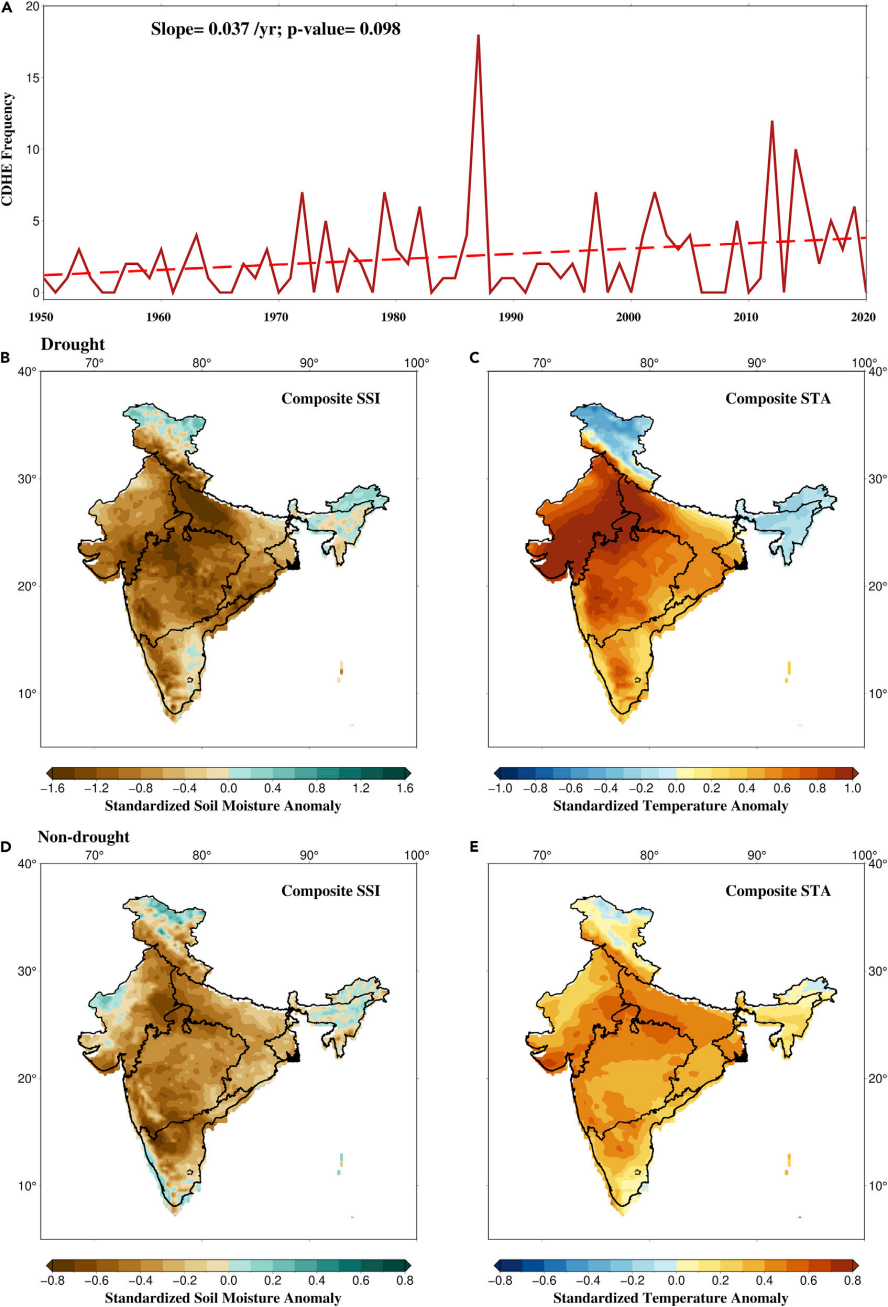
Observed changes in CDHEs during the summer monsoon season in India (A) Annual monsoon CDHE frequency from 1950–2020. A increasing trend (p value=0.098) is observed. (B) Standardized soil moisture (SSI) composite of drought CDHEs. (C) Standardized temperature anomaly (STA) composite of drought CDHEs. (D) Standardized soil moisture (SSI) composite of non-drought CDHEs, and (E) standardized temperature anomaly (STA) composite of non-drought CDHEs.

Next, we used STA and SSI pentads during the summer monsoon season to estimate the annual CDHE frequency during the observed period over India. While previous studies used precipitation to characterize CDHEs (Sharma and Mujumdar, 2017; Mishra et al., 2020; Dash and Maity, 2021; Guntu and Agarwal, 2021), considering the importance of soil moisture variability on land-atmospheric feedbacks in the summer monsoon season, we employ SSI to characterize CDHEs. CDHEs during the summer monsoon increased in the observed period (Figure 2A). The highest frequency of CDHEs (18 pentads) was observed in 1987, which can be attributed to severe drought. Similarly, most years with higher CDHE frequency experienced summer monsoon drought. For instance, 1972 (7 pentads), 1974 (5 pentads), 1979 (7 pentads), 1982 (6 pentads), 2002 (7 pentads), and 2014 (10 pentads) experienced considerable frequency of CDHE events. Droughts contribute to rise in temperature during the summer monsoon season, which can lead to the co-occurrence of concurrent hot and dry extremes. However, CDHEs also occur during relatively shorter (1–4 pentads) non-drought periods. In recent years, an increased frequency of CDHEs in non-drought years has been noted. For instance, 2012, 2015, 2017, and 2019 did not experience a widespread drought but witnessed considerable CDHEs, primarily due to localized droughts and warming. Widespread summer monsoon droughts have declined sharply in the recent decade; therefore, the increased CDHE frequency is primarily due to the rise in non-drought CDHEs. Moreover, the increase in non-drought CDHEs indicates that even if drought occurrence remains unchanged, CDHE frequency will continue to rise (IPCC, 2021).

Subsequently, we examined the spatial variation in temperature extremes and soil moisture deficits over India. We estimated the composite SSI and STA during CDHEs. Since the process driving CDHEs during the drought and non-drought periods is different, we consider them separately. We find a substantial negative SSI over most of India during the drought period (Figure 2B). Almost all the regions exhibit SSI less than −0.8; however, a few regions are affected by SSI less than −1.2. Similarly, in the case of composite STA, we find that almost the entire Northwest region is affected by standardized anomaly higher than 0.8 (Figure 2C). Our results show that majority of the country is affected by soil moisture deficits and extreme temperatures during the non-drought period. However, compared to the drought period, the intensity of SSI and STA is considerably less. The composite STA during non-drought period shows a considerable overlap with the areas with soil moisture deficits implying that those regions are affected by CDHEs (Figures 2D and 2E). Overall, central north-India (20–30N, 75–85E) is identified as a hotspot of CDHEs in both the drought and non-drought periods. The difference in intensity during the drought and non-drought periods can be because of the variations in land-atmospheric coupling during these periods.

### Occurrence of compound extremes during drought and non-drought periods

**Figure 3:**
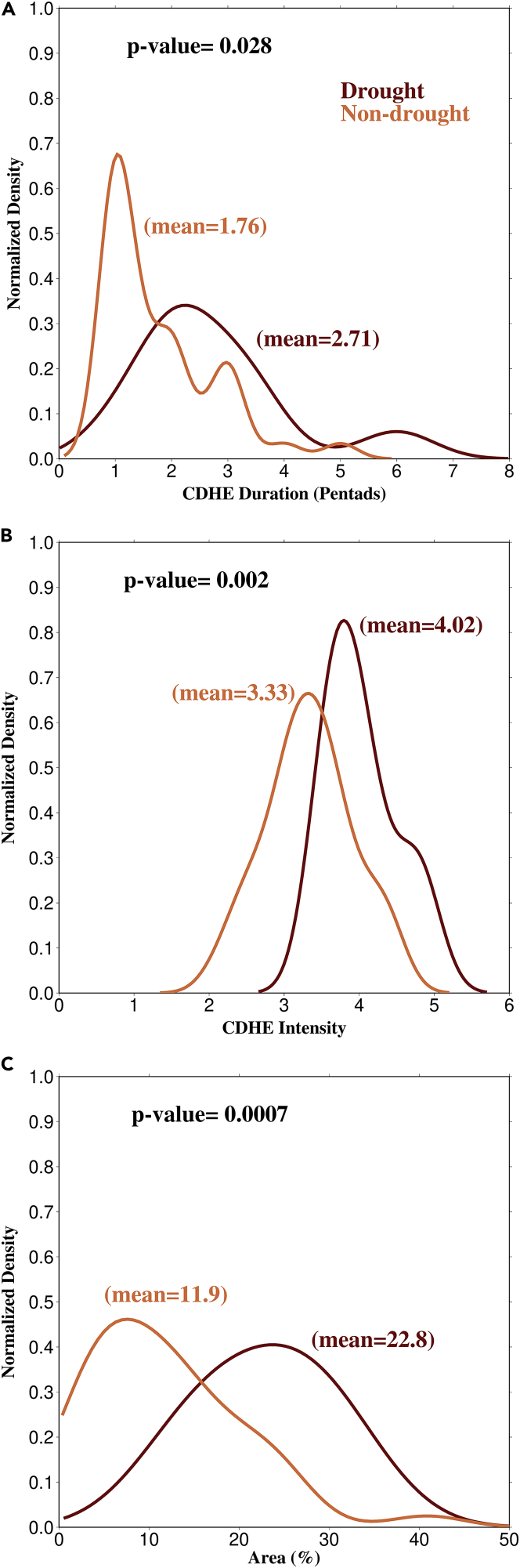
Compound extremes characteristics during drought and non-drought periods Normalized densities of CDHE (A) duration, (B) intensity, and (C) area (%) for the drought (red) and non-drought (orange) periods using kernel-density estimates. All three variables exhibit significant (p value<0.05) differences between the two periods. The mean values for each variable are provided.

As the characteristics of CDHEs during droughts and non-droughts vary, we examined the differences based on their duration, intensity, and areal coverage. We find a significant (p-value < 0.05) difference in duration of compound extremes during drought and non-drought periods (Figure 3A). For instance, mean duration of CDHEs during droughts is longer (2.71 pentads) than non-drought periods (1.76 pentads). While most non-drought CDHEs have a duration of only one pentad, CDHE duration during drought is more spread out, with some events lasting about six pentads. The longer duration of CDHEs during drought can be due to the land-atmospheric feedback, where the dry extremes lead to hot extremes and warm spells (Miralles et al., 2019; Vogel et al., 2021). Since summer monsoon droughts occur due to low rainfall, the soil moisture is not rejuvenated for a prolonged period indicating a longer dry period. The long dry period and land-atmospheric feedback result in an increased warm spell frequency, which can lead to longer CDHE durations (Manning et al., 2019). On the other hand, during non-drought years, dry periods occur due to breaks (dry spells) in monsoon rainfall, which deplete soil moisture. However, these dry periods do not exist for an extended period as the dry spell durations are shorter. Dry spell frequency has increased in the observed period (Singh et al., 2014), which could be a reason for the rise in non-drought CDHEs.

Another effect of the land-atmospheric coupling is the difference in intensity between CDHEs in drought and non-drought periods. CDHEs have significantly (p-value < 0.05) higher intensities during droughts than during non-drought periods (Figure 3B). For instance, mean intensity of CDHEs during droughts is four pentads, whereas, while during non-droughts it is around three pentads. The higher intensity of CDHEs during droughts may be due to the self-intensification mechanism where the dry and hot extremes intensify one another (Miralles et al., 2019; Alizadeh et al., 2020). During droughts, the lack of soil moisture reduces evaporation and partitioning of solar radiation into latent heat, which causes a larger fraction of the incoming radiation to be translated into sensible heat, warming the environment (Fischer et al., 2007a). High temperatures increase evaporative demand, further desiccate soils, and increase the temperature. This cycle of drying and warming impedes the formation of clouds and, in turn, restrains local convective precipitation, further intensifying these extremes (Schumacher et al., 2019; Alizadeh et al., 2020). CDHEs in the non-drought period are primarily associated with dry spells and are preceded by wet spells. Hence, a longer time is required for soil desiccation, limiting the development of hot extremes over a region and prevents high CDHE intensities.

Next, we evaluated the differences in the areal coverage of CDHEs during droughts and non-drought years. The areal coverage of CDHEs reveals that such events affect significantly larger areas (p-value < 0.05) during droughts than during non-drought periods (Figure 3C). For non-drought CDHEs, most events have smaller coverage with a mean area of 11.9% of the country. On the other hand, CDHEs during drought have a mean areal coverage of 22.8%, with most such events covering larger areas than non-drought CDHEs. The significant difference in the areal coverage can be attributed to another mechanism associated with the land-atmospheric feedback, i.e., self-propagation. During self-propagation, dry and hot extremes are spread from one region to downwind regions (Herrera-Estrada et al., 2019; Miralles et al., 2019; Schumacher et al., 2022). During droughts, when evaporation is reduced due to the lack of soil moisture, the atmospheric supply of water is depleted, and this deficit may propagate in time and space (Schumacher et al., 2022), causing CDHEs to spread over a vast area. In the case of the non-drought period, the dry spells that drive CDHEs have a shorter duration as they are preceded and followed by high intensity wet episodes (Rajeevan et al., 2010; Singh et al., 2014). The shorter duration of dry spells prevents the complete depletion soil moisture over a particular region, which limits the propagation of CDHEs. Hence, CDHEs during non-drought period have smaller areal coverage.

**Figure 4:**
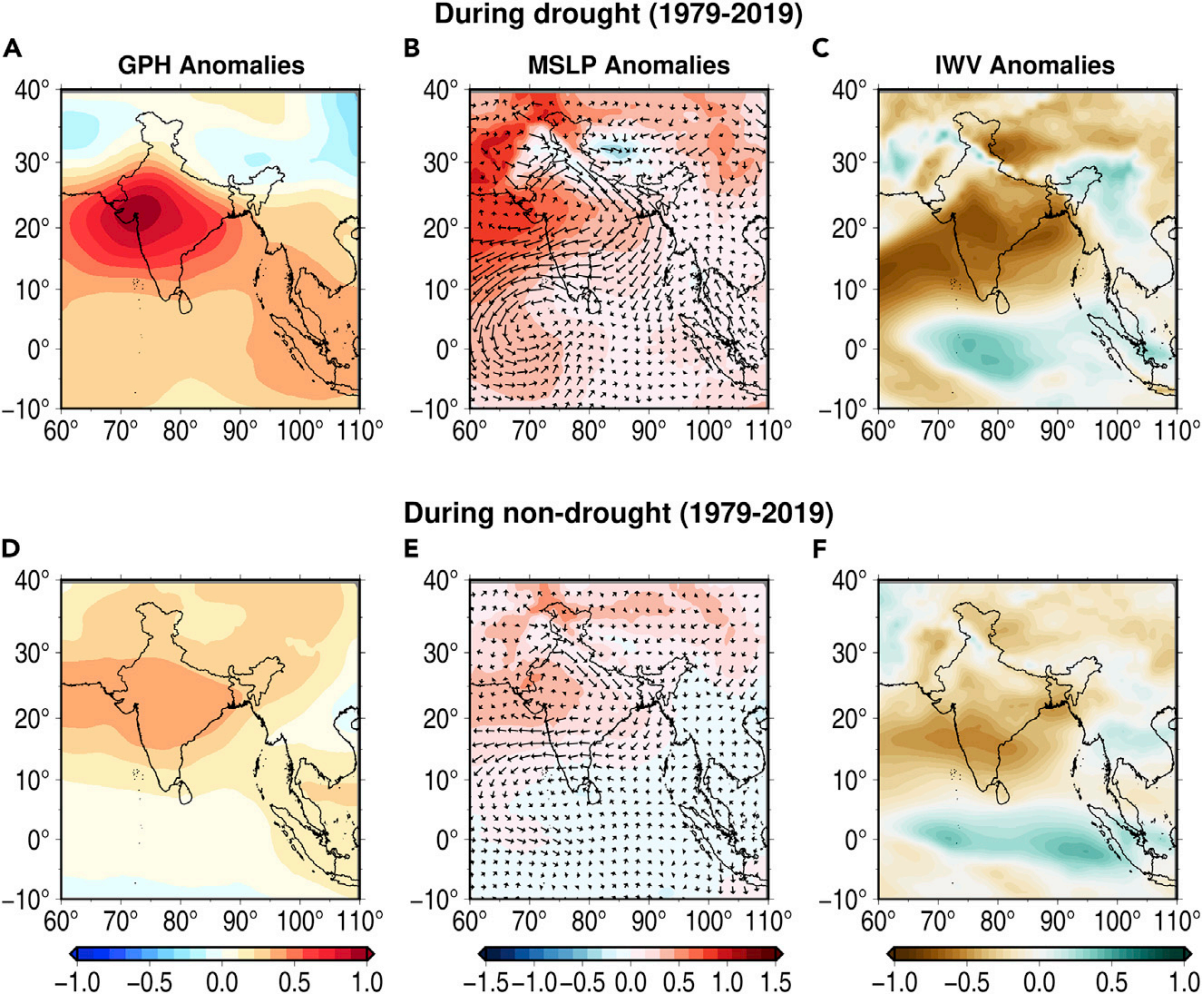
Atmospheric conditions during drought and non-drought periods Composite anomalies of (A) Geopotential Height, (B) Mean Sea Level Pressure (shaded) and wind at 850 hPa (arrows), (C) Integrated water vapor during the drought period. (D), (E), and (F) represent the same but for non-drought period. Anomalies were constructed using the data from ERA5 reanalysis for 1979–2019 period.

The characteristics of CDHEs during drought and non-drought periods are consistent with the atmospheric anomalies (Figure 4). We constructed composites of anomalies of geopotential height, mean sea level pressure, wind (u,v) at 850 hPa, and integrated water vapor for CDHEs that occurred during drought and non-drought periods in the summer monsoon. We find that relatively more intense anomaly composite exist for CDHEs that occurred during drought compared to non-drought periods (Figure 4). Atmospheric anomalies during CDHEs in drought and not drought period exhibit positive geopotential height and mean sea level pressure anomalies while negative integrated water vapor anomalies. The anti-cyclonic wind pattern is also present for CDHEs in drought and non-drought periods. However, the intensity of anomalies during drought is much stronger than during non-drought period (Figure 4).

The occurrence of dry spells directly affects compound extremes during non-drought years as it influences their duration, intensity, and areal coverage. On the other hand, we note a substantial overlap in the characteristics of CDHEs during drought and non-drought, which indicates that dry spells alone may not be the sole cause of non-drought CDHEs. Panda et al. (2017) examined the change in heatwaves and warm spells over India and reported an increase in such events in the recent period. Hence, if dry spells are associated with rising warm spells, the latter can accelerate soil moisture depletion and warming during non-drought years. This accelerated depletion of soil moisture also makes a larger portion of the dry spell period available for intense CDHEs, increasing the duration and areal extent of such extremes.

### Relative contribution of major drivers of CDHE

**Figure 5:**
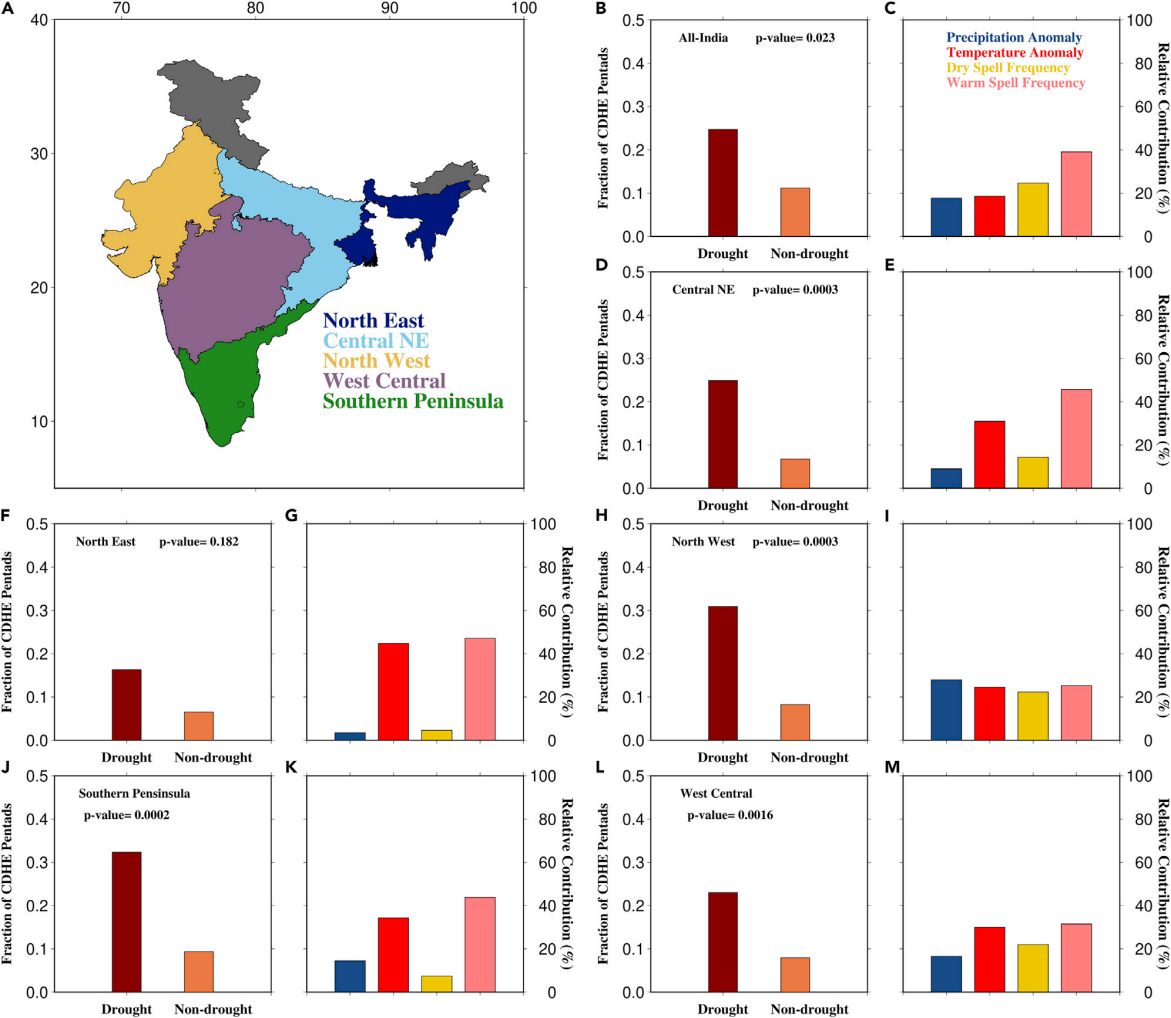
Relative contribution of major drivers of CDHE (A) Homogenous rainfall regions of India, fraction of drought and non-drought pentads in (B) All-India (p value<0.05). (D) Central Northeast (p value<0.05). (F) North East (p value<0.05). (H) North West (p value<0.05). (J) Southern peninsula (p value<0.05). (L) West Central regions (p value<0.05) and Relative contribution of precipitation anomaly, temperature anomaly, dry spell frequency, and warm spell frequency in (C) All-India. (E) Central Northeast. (G) North East. (I) North West. (K) Southern peninsula. (M) West Central regions.

Dry and warm spell frequency plays a substantial role in controlling CDHEs during the summer monsoon. While dry spells are essential for developing non-drought CDHEs, warm spells can affect all CDHE development. Since we consider CDHEs during the summer monsoon period, changes in the monsoon precipitation can also potentially control CDHEs over India. Moreover, recent studies have reported changes in rainfall and temperature as potential drivers of CDHEs (Chiang et al., 2018; Manning et al., 2019; Alizadeh et al., 2020; Bevacqua et al., 2022). Hence, to discern the role of each of these drivers on compound extremes, we estimated their relative contributions. In addition, the fraction of CDHEs formed during drought and non-drought years was also calculated (Figure 5A).

We find that the likelihood of compound extremes is higher during monsoon droughts as the fraction of CDHEs during droughts is more than twice that during non-drought periods over India (Figure 5B). The Northwest and Southern peninsular regions experience most CDHEs in India, as they exhibit the largest fraction of compound extremes during drought and non-drought period (Figures 5H and 5J). In the Northwest region, CDHEs occur 30% of the time during droughts and 8% of the time during non-drought years (Figure 5H). Similarly, the Southern Peninsular region has an elevated fraction during drought (32%) and non-drought (9%) periods (Figure 5J). Despite the similarity in the fraction of compound extremes across India, the primary drivers causing these extremes differ. We find that compound event frequency over India is primarily driven by warm spell and dry spell frequency with the highest relative contribution among the four drivers (Figure 5C). On the other hand, in most regions, temperature during the monsoon season and warm spell frequency are the dominant drivers. For example, compound extreme frequency in the Southern Peninsula is dominated by warm spell frequency (43%) and temperature (34%) [Figure 5K]. However, the Northwest and West Central regions deviate from this as all drivers show similar contributions (Figures 5I and 5M).

The influence of warm spells and monsoon temperatures on CDHE over India implies that rather than changes in the summer monsoon precipitation, the rising temperatures drive CDHE occurrence. Recent studies have examined the primary drivers of CDHEs and found that such events are increasingly being driven by long-term warming (Chiang et al., 2018; Manning et al., 2019; Alizadeh et al., 2020). In recent years, India witnessed increased warm spells and heatwaves (Panda et al., 2017) leading to increased frequency and intensity of all CDHEs (Sharma and Mujumdar, 2017; Mishra et al., 2020; Guntu and Agarwal, 2021). Moreover, Bevacqua et al. (2022) showed that such a rise in future warm spell frequency can cause the future CDHEs occurrence to depend on mean precipitation trends as future dry extremes will always coincide with these warm spells.

### The role of land-atmospheric coupling on CDHEs

We find that the dominant driver of CDHEs in India is warm spell frequency; however, there are considerable variations in the contribution of these drivers across different regions. The fraction of CDHEs during drought and non-drought periods varies in different parts of the country. A potential cause for these disparities may be the differences in land-atmospheric feedback. Moreover, we find that CDHE frequency, intensity, and coverage are also linked to the land-atmospheric coupling. Therefore, to assess the role of land-atmospheric feedback on CDHEs over India, we estimated the composite soil moisture-temperature (SM-T) coupling over the region. We considered years without CDHEs as normal years (N), years with CDHEs during drought years (D), and non-drought years (ND).

**Figure 6:**
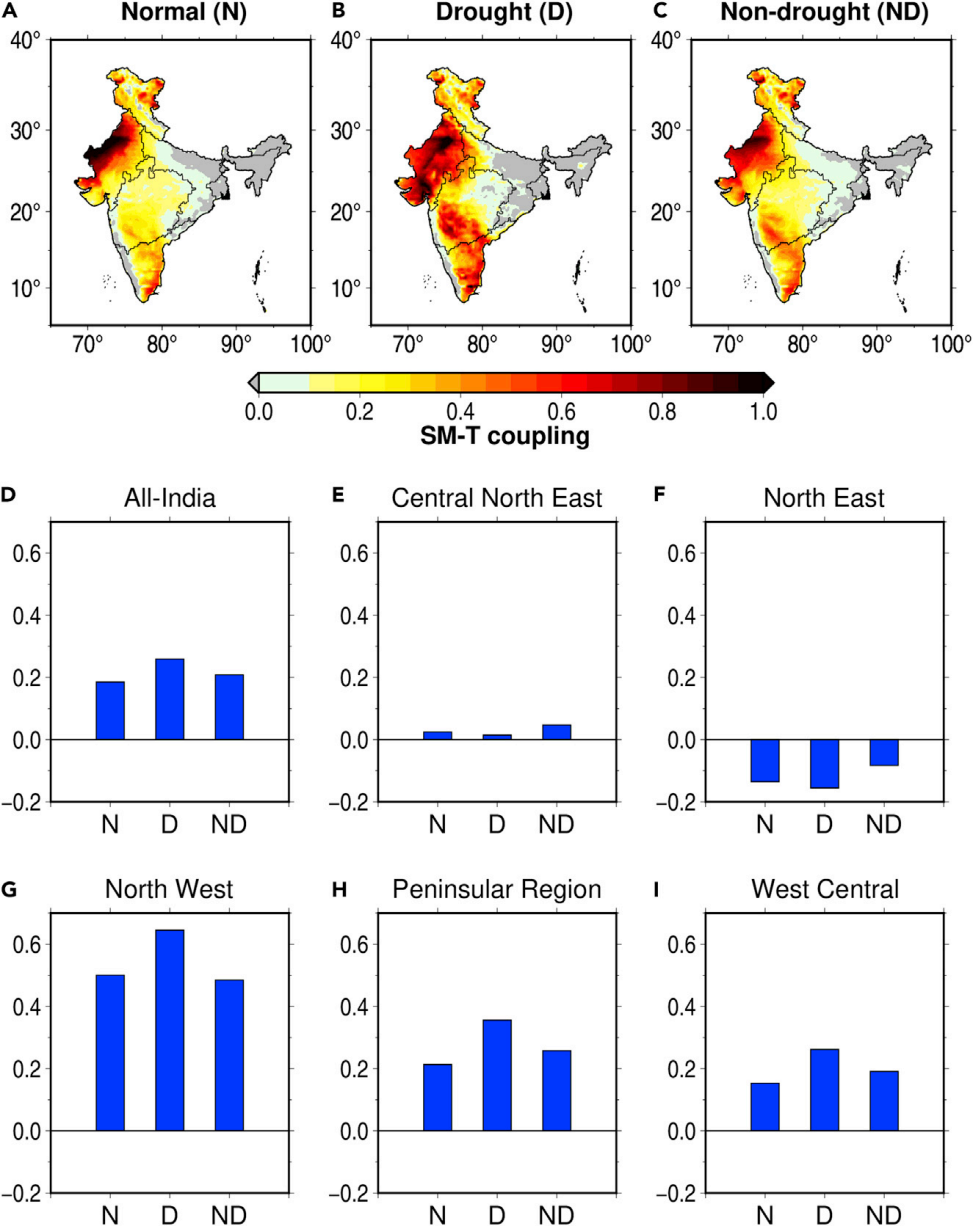
Land-atmospheric coupling over the Indian region Soil moisture-temperature coupling composite during (A) normal, (B) drought, and (C) non-drought years, and spatially averaged soil moisture for (D) All-India, (E) Central Northeast, (F) North East, (G) North West, (H) Southern peninsula, (I) West Central. Here values closer to 1 imply strong coupling and 0 or lower signify no coupling.

During normal years, the highest coupling is observed in the Northwest region (Figures 6A and 6G) followed by the Southern peninsula (Figure 6H). Ramarao et al. (2016) reported that the strong coupling in these regions as evapotranspiration is largely determined by the degree of soil moisture deficit rather than net radiation. On the contrary, the central-northeast and Northeast regions exhibit weak land-atmospheric coupling, which can be attributed to limited energy to drive evapotranspiration (Ramarao et al., 2016) [See also aridity variation in Figure S2]. During droughts, these high and low coupling patterns intensify and extend over larger areas (Figure 6B). On the other hand, the coupling strength is comparatively weaker during non-drought periods (Figure 6C). The spatially averaged SM-T coupling over India shows that normal years have the lowest coupling while drought years exhibit the highest coupling (Figure 6D).

The differences in SM-T coupling during drought and non-drought periods are reflected in the occurrence of compound extremes. The stronger SM-T coupling during droughts can drive up CDHE frequency during the period. The soil moisture deficits during droughts cause the incoming shortwave radiation to be converted to sensible heat driving up the local temperature. During the non-drought period, incoming shortwave radiation is converted primarily to latent heat because of the evaporation of soil moisture, preventing temperature extremes and subsequently compound extremes. Furthermore, areas having strong coupling have a higher frequency of CDHEs. For example, the Northwest and Southern regions where SM-T coupling is the strongest are also the regions that exhibit the largest fraction of compound extremes. The lack of soil moisture in these regions aid in the self-intensification of land-atmospheric feedback causing intense CDHEs.

While SM-T coupling directly affects CDHEs, it also indirectly influences their drivers. In the Northwest region, there is a substantial contribution from precipitation anomaly and dry spell frequency compared to other regions (Figure 5I). Since this region is arid, the soil moisture is strongly coupled with precipitation. The strong coupling over this region can cause any fluctuations in soil moisture to impact the local temperature and trigger CDHEs. However, a similar pattern does not occur in the Southern peninsular region despite the strong coupling. The relatively weaker SM-T coupling in this area means that precipitation-driven changes in soil moisture do not entirely influence the regional temperature and could account for the relative contribution of its drivers. Finally, the lack of SM-T coupling in the Northeast region means that increase in CDHE frequency is solely due to regional warming trends, which affect monsoon temperature anomalies and warm spell frequency.

### Discussion and conclusions

Considering CDHE hazards, particularly during the monsoon, it is essential to quantify the primary drivers of such extremes in India. Our results show that despite an increase in monsoon period soil moisture over India, CDHE frequency has increased during the observed record of 1950–2020, which is consistent with previous studies over India (Sharma and Mujumdar, 2017; Dash and Maity, 2021; Guntu and Agarwal, 2021). A higher annual frequency of CDHEs is associated with drought years; however, in the recent decade, there has been a rise in CDHE frequency in non-drought years. This rise in the frequency of non-drought CDHEs could be due to a decline in monsoon rainfall in recent years, which has led to an increase in dry spell frequency (Singh et al., 2014; Ramarao et al., 2016). The influence of dry spells, which are less intense than droughts, is reflected in the duration, intensity, and areal extent of CDHEs. We find that the drought period CDHEs are more prolonged, more intense, and cover a larger area than non-drought CDHEs due to substantial land-atmospheric feedbacks (Miralles et al., 2019). Subsequently, the rise in warm spell frequency over India driven by anthropogenic warming (Panda et al., 2017) can also impact CDHEs by modulating the SM-T coupling over a region. This influence is also evident in our results which show that CDHE frequency in most regions is linked mainly to warm spell frequency and monsoon temperature anomalies. This pattern of CDHEs being increasingly driven by rising temperatures has been observed in various regions of the globe (Chiang et al., 2018; Alizadeh et al., 2020; Vogel et al., 2021). The rise in warming-driven CDHE frequency signifies that even if droughts remain unaffected, CDHEs will continue to rise (IPCC, 2021). Furthermore, consistent with previous studies (Seneviratne et al., 2010; Miralles et al., 2012,^2019^; Alizadeh et al., 2020), we find that land-atmospheric coupling influences CDHE frequency directly, by modulating the local soil moisture and temperature, and indirectly, by impacting their drivers.

Our analysis is based on ERA5 reanalysis data instead of station-based observations. ERA5 reanalysis provides the land-atmospheric variables through coupled land-atmosphere simulations that can be used to examine the role of land-atmospheric coupling. Despite the ERA5 reanalysis performs better than other high-resolution reanalysis products, there can be differences in trends in land-atmospheric variables compared to observations. For instance, the summer monsoon season drying trend over the Indo-Gangetic Plain is relatively underestimated in ERA5 reanalysis compared to observations (Mishra, 2016, Shah and Mishra, 2016). Moreover, our analysis is based on a fixed threshold of soil moisture and temperature to estimate compound dry-hot extremes. The identified frequency of compound extremes can change with the choice of threshold. Notwithstanding these limitations, our findings showing the differences in compound hot-dry extremes during the drought and non-drought summer monsoons are important for evaluating the impacts of these extremes on other sectors (water resources and agriculture). Moreover, estimation of hot-day compound extremes based on rainfall and temperature may not account for the role of land-atmosphere coupling. Considering the importance of compound extremes in India, it is vital to develop early warning systems to predict these events.

Based on our findings, we conclude the following:

1) CDHE frequency over India has increased during the observed period (1950–2020). While majority of CDHEs occurred during droughts caused by monsoon failures, the recent rise in compound event frequency is primarily due to increase in CDHEs during non-drought years.
2) The rise in compound dry-hot extremes during non-drought years can be attributed to the decline in summer monsoon rainfall as it led to an increase in dry spells during this period. Additionally, a warming-driven rise in warm spells over India is another major driver of compound extremes. While dry spells and droughts lead to the rise frequency of CDHEs, the increase in warm spell frequency further intensifies such extremes.
3) Compared to non-drought period, compound extremes during droughts have higher intensity, duration, and areal extent making such events extremely hazardous. This increased severity of CDHEs during droughts is due to the stronger land-atmospheric coupling.
4) While the land-atmospheric coupling influences the compound extremes directly by modulating soil moisture and temperature, the coupling can also affect the drivers of these extremes causing regional differences in CDHE occurrence.

## STAR★Methods

### Key resources table

**Table:** 

REAGENT OR RESOURCE	SOURCE	IDENTIFIER
**Deposited data**		

ERA5 reanalysis data	ECMWF	https://doi.org/10.24381/cds.adbb2d47

**Software and algorithms**		

Code to reproduce the results of this study	Authors	https://github.com/rajeevakshay/CDHE_India

### Resource availability

#### Lead contact

Further information and requests for resources should be directed to the lead contact, V. Mishra (vmishra@iitgn.ac.in)

#### Materials availability

This study did not generate new datasets.

### Method details

#### Data

We obtained hourly total precipitation, air temperature at 2m height, dew-point temperature, wind, surface pressure, humidity, and volumetric soil moisture for the top three layers (0-7cm, 7–28cm, 28–100cm) at a 31km spatial resolution from the ERA5 reanalysis, which perform better than the other reanalysis products over India (Mahto and Mishra, 2019). Unlike off-line simulations from land surface models, coupled simulations from the ERA5 reanalysis can be used to examine the land-atmospheric coupling. We used the hourly variables from ERA5 to estimate daily accumulated precipitation, maximum temperature, and volumetric soil moisture for each layer. Moreover, we obtained daily temperature, dew-point temperature, wind, surface pressure, and humidity from the ERA5 reanalysis, which were used to estimate potential evapotranspiration (PET) using the Penman-Monteith method (Penman, 1948; Zotarelli et al., 2010). We used the preliminary ERA5 reanalysis from 1950 to 1978 and the final release for 1979 to 2020 (Hersbach et al., 2020). The availability of continuous data of atmospheric and land-surface variables at high temporal and spatial resolutions is vital for examining CDHEs (Hirschi et al., 2010). We estimated the top 60cm to evaluate soil moisture content using volumetric soil moisture from ERA5 as it is the effective root-zone depth for most crops (Mishra et al., 2018).

#### Identification of CDHEs

We averaged the daily maximum temperature and soil moisture (root zone) over five days to convert to pentads. We calculated the standardized temperature anomaly (STA) and standardized soil moisture index (SSI) using the maximum temperature and soil moisture pentads from ERA5, respectively. We only considered the pentads for the summer monsoon season (June-September, JJAS) in our analysis. While previous studies used copula (Zhou and Park Williams, 2019; Zhou and Zhang, 2019) to identify the compound extremes, we adopted a threshold-based approach that has been extensively used to examine compound extremes in India (Sharma and Mujumdar, 2017; Mishra et al., 2020; Ambika and Mishra, 2021; Dash and Maity, 2021; Guntu and Agarwal, 2021). Since the pentad-based estimates smooth out short-term fluctuations in soil moisture and temperature, compound extremes were identified using the fixed thresholds for pentads. We consider pentads for which STA exceeds 1 as hot extremes and pentads with SSI less than −1 as dry extremes. STA (SSI) of 1(-1) corresponds approximately to the 80^th^ (20^th^) percentile of maximum temperature (soil moisture). Therefore, pentads where hot and dry extremes co-occur, i.e., STA>= 1 and SSI<= −1, were identified as CDHE.

Since we focus on the summer monsoon droughts, we only considered pentads during the monsoon season each year. We used the spatially averaged soil moisture overIndia as our aim was to identify large scale CDHE during the summer monsoon season that have large impacts. However, the analysis can be also performed using soil moisture for different regions to examine regions CDHEs. We estimated the standardized soil moisture index (SSI) using the soil moisture anomaly derived from mean soil moisture for the summer monsoon season. Finally, we considered years with SSI less than −1 as drought years. We estimated the fraction of CDHEs during drought and for those which are not drought driven. The fraction of CDHEs during drought is calculated by dividing the total CDHEs pentads that occurred during drought years by the total drought pentads. For example, if there are ten drought years, the total drought pentads are 250 (25 pentads during monsoon). Then if out of 250, 25 pentads had compound extremes, the fraction of CDHEs during drought is 0.1. In the case of the non-drought period, all years in which droughts did not occur were considered non-drought years. In this case, the fraction of CDHEs during the non-drought period is the ratio of total CDHEs pentads that occurred during non-drought years to the total non-drought pentads. Subsequently, we aggregated the fraction of CDHEs during the two periods spatially across the different homogenous rainfall regions of India and examined the major drivers of CDHEs.

#### Estimation of dry and warm spells

The Indian region is affected by dry and wet spells associated with shifts in the continental tropical convergence zone (Annamalai and Slingo, 2001). These dry and wet spells in the summer monsoon season can be caused by variability in the seasonal monsoon rainfall (Rajeevan et al., 2010). Moreover, as precipitation variability affects soil moisture conditions, dry and wet spells can strongly influence the occurrence of CDHEs during the monsoon period. We used detrended precipitation anomalies to define the dry and wet spells to exclude the influence of seasonal mean precipitation trends. We first removed the time varying mean from the daily precipitation time series and normalize those anomalies by the standard deviation of precipitation over the entire record (1950–2020). Finally, we define dry/wet spells as events having at least three consecutive days with precipitation anomalies consistently exceeding one standard deviation of daily precipitation. Previous studies have used one standard deviation (Rajeevan et al., 2010; Singh et al., 2014) and 0.7 standard deviation (Mandke et al., 2007) as thresholds to identify dry spells. We estimated the annual dry spell frequency using the total number of dry spells during the summer monsoon period.

Globally, due to land-atmospheric coupling, dry spells are associated with warm periods (Miralles et al., 2019). These warm spells influence the intraseasonal variations of the Indian summer monsoon by modulating soil moisture and can further exacerbate impacts of droughts (Mazdiyasni and AghaKouchak, 2015; Ramarao et al., 2016). We used daily maximum temperature from ERA5 to characterize the warm spells as warm spells can affect CDHE occurrence by controlling the land-atmosphere coupling. We define a warm spell as the 3-day exceedance of the 90^th^ percentile threshold of the daily maximum temperature values of the entire period (Mazdiyasni and AghaKouchak, 2015; Panda et al., 2017). We calculated the annual frequency of warm spells by estimating the total number of warm spells during the summer monsoon season.

#### Land-atmospheric coupling

We estimated the feedback between soil moisture (SM) and temperature (T) as a measure of land-atmospheric coupling through the differential skill of two energy balance approaches based on evapotranspiration (ET) and potential evapotranspiration (PET) [Miralles et al., 2012, 2014]. The land-atmospheric coupling framework was initially developed for the annual timescale. However, the framework can be applied for shorter timescale, such as during heatwaves, by considering standardized anomalies rather than climatological mean (Miralles et al., 2012,^2014^). We estimated the SM-T coupling matrix (π) at pentad scale, which is defined as:

(Equation 1) $$\pi =T^{\prime }\left({H_{et}}^{\prime }-{H_{pet}}^{\prime }\right)$$

(Equation 2) $${H_{et}}^{\prime }=R_{n}-\lambda ET$$

(Equation 3) $${H_{pet}}^{\prime }=R_{n}-\lambda PET$$

Where, *T* is the temperature, *H*_*et*_ and *H*_*pet*_ are the sensible heat flux terms for ET and PET, respectively. *R*_*n*_ is the surface net radiation in Wm^−2^. The primes in *T*, *H*_*et*_ and *H*_*pet*_ indicate the respective standardized anomaly. The latent heat of vaporization λ (Jkg^−1^) can be estimated as a function of *T* (Henderson-Sellers, 1984), while π can be positive or negative. Negative values of π represent no coupling, whereas coupling strength increases with increasing positive values. Positive values of π are found in the regions where a large fraction of variability in *T* is explained by the partitioning of latent and sensible heat under soil moisture deficit (Miralles et al., 2012).

The units of ET and PET are in kgm^−2^sec ^−1^while estimating both the energy terms in Equations 2 and3. The SM-T coupling consists of two terms: temperature ($T^{\prime }$) and energy $\left({H_{et}}^{\prime }-{H_{pet}}^{\prime }\right)$, where energy term describes the effect of declining soil moisture on the energy budget by providing negative feedback to temperature through increasing sensible heat, especially during the compound drought and heatwave condition. We estimated the land-atmospheric coupling matrix (π) for the observed period 1950–2020 over the Indian region using the variables (*T*, *R*_*n*_, *H*_*et*_ and *H*_*pet*_) from ERA5 reanalysis.

## Data Availability

• This paper analyzes existing, publicly available data. These accession numbers for the datasets are listed in the key resources table. • All original code has been deposited on Github and is publicly available. Links are listed in the key resources table. • Any additional information required to reanalyze the data reported in this paper is available from the lead contact upon request.

## References

[bib1] Aadhar S., Mishra V. (2020). Increased drought risk in South Asia under warming climate: implications of uncertainty in potential evapotranspiration estimates. J. Hydrometeorol..

[bib2] Alizadeh M.R. (2020). A century of observations reveals increasing likelihood of continental-scale compound dry-hot extremes. Sci. Adv..

[bib3] Allen C.D., Macalady A.K., Chenchouni H., Bachelet D., McDowell N., Vennetier M., Kitzberger T., Rigling A., Breshears D.D., Hogg E.T. (2010). A global overview of drought and heat-induced tree mortality reveals emerging climate change risks for forests. For. Ecol. Manag..

[bib70] Ambika A.K., Mishra V. (2019). Observational evidence of irrigation influence on vegetation health and land surface temperature in India. Geophysical Research Letters.

[bib4] Ambika A.K., Mishra V. (2021). Modulation of compound extremes of low soil moisture and high vapor pressure deficit by irrigation in India. Geophys. Res. Atmos..

[bib5] Annamalai H., Slingo J.M. (2001). Active/break cycles: diagnosis of the intraseasonal variability of the asian summer monsoon. Clim. Dynam..

[bib6] Berg A., Findell K., Lintner B., Giannini A., Seneviratne S.I., van den Hurk B., Lorenz R., Pitman A., Hagemann S., Meier A. (2016). Land–atmosphere feedbacks amplify aridity increase over land under global warming. Nat. Clim. Chang..

[bib7] Bevacqua E., Zappa G., Lehner F., Zscheischler J. (2022). Precipitation trends determine future occurrences of compound hot–dry events. Nat. Clim. Chang..

[bib8] Bollasina M.A., Ming Y., Ramaswamy V. (2011). Anthropogenic aerosols and the weakening of the south asian summer monsoon. Science.

[bib9] Chiang F., Mazdiyasni O., AghaKouchak A. (2018). Amplified warming of droughts in southern United States in observations and model simulations. Sci. Adv..

[bib10] Dash S., Maity R. (2021). Revealing alarming changes in spatial coverage of joint hot and wet extremes across India. Sci. Rep..

[bib11] Fischer E.M., Seneviratne S.I., Vidale P.L., Lüthi D., Schär C. (2007). Soil moisture–atmosphere interactions during the 2003 European summer heat wave. J. Clim..

[bib12] Fischer E.M., Seneviratne S.I., Lüthi D., Schär C. (2007). Contribution of land-atmosphere coupling to recent European summer heat waves. Geophys. Res. Lett..

[bib13] Garg S., Mishra V. (2019). Role of extreme precipitation and initial hydrologic conditions on floods in godavari river basin, India. Water Resour. Res..

[bib14] Guntu R.K., Agarwal A. (2021). Disentangling increasing compound extremes at regional scale during Indian summer monsoon. Sci. Rep..

[bib15] Hauser M., Orth R., Seneviratne S.I. (2016). Role of soil moisture versus recent climate change for the 2010 heat wave in western Russia. Geophys. Res. Lett..

[bib16] Henderson-Sellers B. (1984). A new formula for latent heat of vaporization of water as a function of temperature. Q. J. R. Meteorol. Soc..

[bib17] Herrera-Estrada J.E., Martinez J.A., Dominguez F., Findell K.L., Wood E.F., Sheffield J. (2019). Reduced moisture transport linked to drought propagation across north America. Geophys. Res. Lett..

[bib18] Hersbach H., Bell B., Berrisford P., Hirahara S., Horányi A., Muñoz-Sabater J., Nicolas J., Peubey C., Radu R., Schepers D. (2020). The ERA5 global reanalysis. Q. J. R. Meteorol. Soc..

[bib19] Hettiarachchi S., Wasko C., Sharma A. (2022). Do longer dry spells associated with warmer years compound the stress on global water resources?. Earth's Future.

[bib20] Hirschi M., Seneviratne S.I., Alexandrov V., Boberg F., Boroneant C., Christensen O.B., Formayer H., Orlowsky B., Stepanek P. (2010). Observational evidence for soil-moisture impact on hot extremes in southeastern Europe. Nat. Geosci..

[bib21] IPCC (2021). Sixth Assessment Report, Global Warming of 1.5°C. An IPCC Special Report on the Impacts of Global Warming of 1.5°C above Pre-industrial Levels and Related Global Greenhouse Gas Emission Pathways, in the Context of Strengthening the Global Response to the Threat of Climate Change. https://www.ipcc.ch/report/ar6/wg1/%0Ahttps://www.ipcc.ch/report/ar6/wg1/%0Ahttps://www.ipcc.ch/report/ar6/wg1/%0Ahttps://www.ipcc.ch/report/ar6/wg1/downloads/report/IPCC_AR6_WGI_Headline_Statements.pdf%0Ahttps://www.ipcc.ch/report/ar6/wg1/%0Ahttps://www.

[bib66] Kumar K. Niranjan (2013). On the observed variability of monsoon droughts over India. Weather and Climate Extremes.

[bib24] Leonard M., Westra S., Phatak A., Lambert M., van den Hurk B., McInnes K., Risbey J., Schuster S., Jakob D., Stafford-Smith M. (2014). A compound event framework for understanding extreme impacts. WIREs Climate Change.

[bib67] Mahto S.S., Mishra V. (2019). Does ERA-5 outperform other reanalysis products for hydrologic applications in India?. Journal of Geophysical Research: Atmospheres.

[bib25] Mandke S.K., Sahai A.K., Shinde M.A., Joseph S., Chattopadhyay R. (2007). Simulated changes in active/break spells during the Indian summer monsoon due to enhanced CO2 concentrations: assessment from selected coupled atmosphere–ocean global climate models. Int. J. Climatol..

[bib26] Manning C., Widmann M., Bevacqua E., Van Loon A.F., Maraun D., Vrac M. (2019). Increased probability of compound long-duration dry and hot events in Europe during summer (1950–2013). Environ. Res. Lett..

[bib27] Mazdiyasni O., AghaKouchak A. (2015). Substantial increase in concurrent droughts and heatwaves in the United States. Proc. Natl. Acad. Sci. USA.

[bib28] Miralles D.G., van den Berg M.J., Teuling A.J., de Jeu R.A.M. (2012). Soil moisture-temperature coupling: a multiscale observational analysis. Geophys. Res. Lett..

[bib29] Miralles D.G., Teuling A.J., van Heerwaarden C.C., Vilà-Guerau de Arellano J. (2014). Mega-heatwave temperatures due to combined soil desiccation and atmospheric heat accumulation. Nat. Geosci..

[bib30] Miralles D.G., Gentine P., Seneviratne S.I., Teuling A.J. (2019). Land–atmospheric feedbacks during droughts and heatwaves: state of the science and current challenges. Ann. N. Y. Acad. Sci..

[bib31] Mishra V., Smoliak B.V., Lettenmaier D.P., Wallace J.M. (2012). A prominent pattern of year-to-year variability in Indian Summer Monsoon Rainfall. Proc. Natl. Acad. Sci. USA.

[bib69] Mishra V. (2016). On the frequency of the 2015 monsoon season drought in the indo-gangetic plain. Geophysical Research Letters.

[bib32] Mishra V., Shah R., Azhar S., Shah H., Modi P., Kumar R. (2018). Reconstruction of droughts in India using multiple land-surface models (1951-2015). Hydrol. Earth Syst. Sci..

[bib33] Mishra V., Thirumalai K., Singh D., Aadhar S. (2020). Future exacerbation of hot and dry summer monsoon extremes in India. npj Clim. Atmos. Sci..

[bib34] Mueller B., Seneviratne S.I. (2012). Hot days induced by precipitation deficits at the global scale. Proc. Natl. Acad. Sci. USA.

[bib35] Mukherjee S., Mishra A.K. (2021). Increase in compound drought and heatwaves in a warming world. Geophys. Res. Lett..

[bib36] Mukherjee S., Ashfaq M., Mishra A.K. (2020). Compound drought and heatwaves at a global scale: the role of natural climate variability-associated synoptic patterns and land-surface energy budget anomalies. J. Geophys. Res. Atmos..

[bib38] Niyogi D., Kishtawal C., Tripathi S., Govindaraju R.S. (2010). Observational evidence that agricultural intensification and land use change may be reducing the Indian summer monsoon rainfall. Water Resour. Res..

[bib39] Panda D.K., AghaKouchak A., Ambast S.K. (2017). Increasing heat waves and warm spells in India, observed from a multiaspect framework. J. Geophys. Res. Atmos..

[bib40] Patel P., Thakur P.K., Aggarwal S.P., Garg V., Dhote P.R., Nikam B.R., Swain S., Al-Ansari N. (2022). Revisiting 2013 Uttarakhand flash floods through hydrological evaluation of precipitation data sources and morphometric prioritization. Geomatics, Nat. Hazards Risk.

[bib41] Penman H.L. (1948). Natural evaporation from open water, bare soil and grass. Proc. R. Soc. Lond. A Math. Phys. Sci..

[bib42] Poumadère M., Mays C., Le Mer S., Blong R. (2005). The 2003 heat wave in France: dangerous climate change here and now. Risk Anal..

[bib43] Rajeevan M., Gadgil S., Bhate J. (2010). Active and break spells of the Indian summer monsoon. J. Earth Syst. Sci..

[bib44] Ramarao M.V.S., Sanjay J., Krishnan R. (2016). Modulation of summer monsoon sub-seasonal surface air temperature over India by soil moisture-temperature coupling. Mausam.

[bib45] Roxy M.K., Ritika K., Terray P., Murtugudde R., Ashok K., Goswami B.N. (2015). Drying of Indian subcontinent by rapid Indian Ocean warming and a weakening land-sea thermal gradient. Nat. Commun..

[bib46] Sarhadi A., Ausín M.C., Wiper M.P., Touma D., Diffenbaugh N.S. (2018). Multidimensional risk in a nonstationary climate: joint probability of increasingly severe warm and dry conditions. Sci. Adv..

[bib47] Schumacher D.L., Keune J., van Heerwaarden C.C., Vilà-Guerau de Arellano J., Teuling A.J., Miralles D.G. (2019). Amplification of mega-heatwaves through heat torrents fuelled by upwind drought. Nat. Geosci..

[bib48] Schumacher D.L., Keune J., Dirmeyer P., Miralles D.G. (2022). Drought self-propagation in drylands due to land–atmosphere feedbacks. Nat. Geosci..

[bib49] Sedlmeier K., Feldmann H., Schädler G. (2018). Compound summer temperature and precipitation extremes over central Europe. Theor. Appl. Climatol..

[bib50] Seneviratne S.I., Lüthi D., Litschi M., Schär C. (2006). Land–atmosphere coupling and climate change in Europe. Nature.

[bib51] Seneviratne S.I., Corti T., Davin E.L., Hirschi M., Jaeger E.B., Lehner I., Orlowsky B., Teuling A.J. (2010). Investigating soil moisture–climate interactions in a changing climate: a review. Earth Sci. Rev..

[bib52] Seneviratne S.I. (2012). Changes in Climate Extremes and Their Impacts on the Natural Physical Environment’, Managing The Risks Of Extreme Events And Disasters To Advance Climate Change Adaptation: Special Report Of the Intergovernmental Panel On Climate Change.

[bib53] Shah D. (2021).

[bib68] Shah H.L., Mishra V. (2016). Hydrologic changes in Indian Subcontinental River basins (1901–2012). Journal of Hydrometeorology.

[bib71] Shah D., Mishra V. (2020). Integrated drought index (IDI) for Drought Monitoring and assessment in India. Water Resources Research.

[bib54] Sharma S., Mujumdar P. (2017). Increasing frequency and spatial extent of concurrent meteorological droughts and heatwaves in India. Sci. Rep..

[bib55] Singh D., Tsiang M., Rajaratnam B., Diffenbaugh N.S. (2014). Observed changes in extreme wet and dry spells during the south Asian summer monsoon season. Nat. Clim. Chang..

[bib56] Toreti A., Cronie O., Zampieri M. (2019). Concurrent climate extremes in the key wheat producing regions of the world. Sci. Rep..

[bib57] Vogel J., Paton E., Aich V., Bronstert A. (2021). Increasing compound warm spells and droughts in the Mediterranean Basin. Weather Clim. Extrem..

[bib59] Zhang X., Obringer R., Wei C., Chen N., Niyogi D. (2017). Droughts in India from 1981 to 2013 and implications to wheat production. Sci. Rep..

[bib60] Zhou S., Williams A.P., Berg A.M., Cook B.I., Zhang Y., Hagemann S., Lorenz R., Seneviratne S.I., Gentine P. (2019). Land–atmosphere feedbacks exacerbate concurrent soil drought and atmospheric aridity. Proc. Natl. Acad. Sci. USA.

[bib61] Zhou S., Giannetto M., DeCourcey J., Kang H., Kang N., Li Y., Zheng S., Zhao H., Simmons W.R., Wei H.S. (2019). Projected increases in intensity, frequency, and terrestrial carbon costs of compound drought and aridity events. Sci. Adv..

[bib62] Zotarelli L. (2010). http://fawn.

[bib63] Zscheischler J., Westra S., van den Hurk B.J.J.M., Seneviratne S.I., Ward P.J., Pitman A., AghaKouchak A., Bresch D.N., Leonard M., Wahl T., Zhang X. (2018). Future climate risk from compound events. Nat. Clim. Chang..

[bib64] Zscheischler J., Martius O., Westra S., Bevacqua E., Raymond C., Horton R.M., van den Hurk B., AghaKouchak A., Jézéquel A., Mahecha M.D. (2020). A typology of compound weather and climate events. Nat. Rev. Earth Environ..

[bib65] Zscheischler J., Orth R., Seneviratne S.I. (2017). Bivariate return periods of temperature and precipitation explain a large fraction of European crop yields. Biogeosciences.

