# Supplementary material for: Climate warming and summer monsoon breaks drive compound dry and hot extremes in India
Source: iScience. 2022 Oct 17;25(11):105377. doi: 10.1016/j.isci.2022.105377 (PMC9636558; doi:10.1016/j.isci.2022.105377)
Supplement: Document S1. Figures S1 and S2 [file mmc1.pdf]

iScience, Volume *25*

Supplemental information

Climate warming and summer monsoon breaks drive compound dry and hot extremes in India

Akshay Rajeev, Shanti Shwarup Mahto, and Vimal Mishra

Supplementary Information

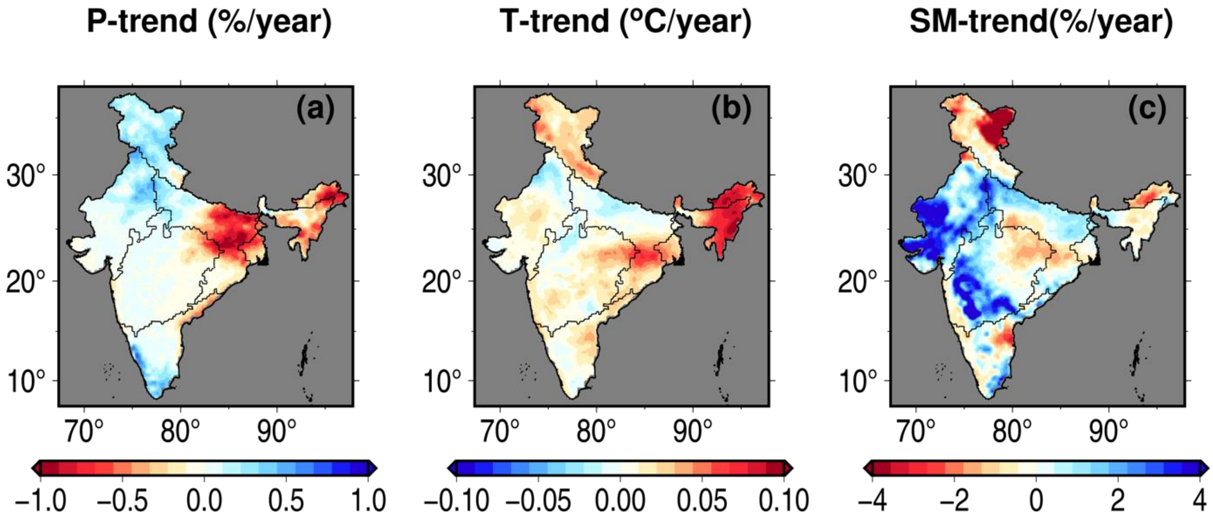

Figure S1: Trend in summer monsoon season a) precipitation (%/year), b) temperature (degree C/year), and c) soil moisture (%/year) for 1950-2020 period, Related to Figure 1.

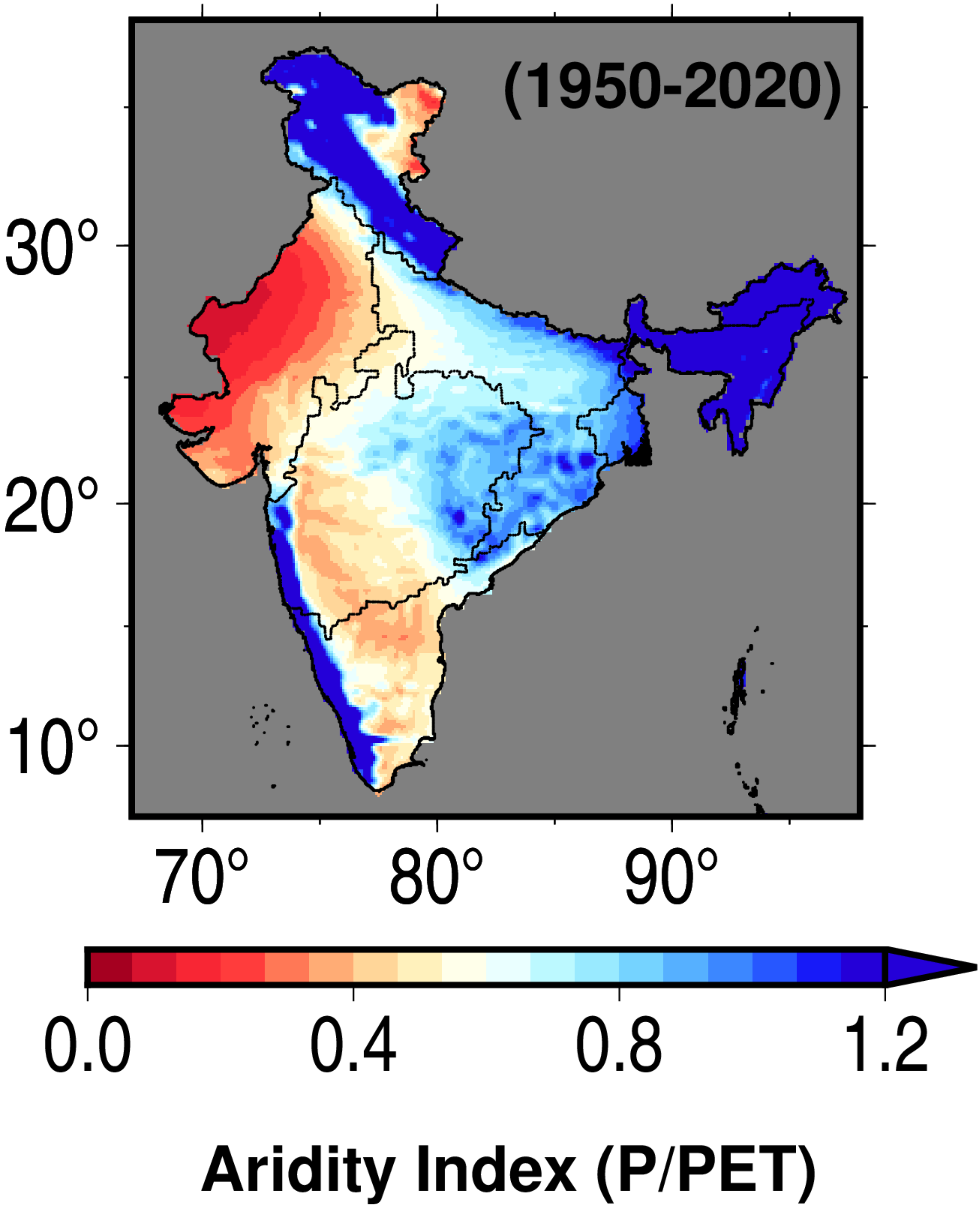

Figure S2: Regional differences in aridity in the various homogenous rainfall regions in India, Related to Figures 5 and 6.
